# Bioactive Peptides and Other Immunomodulators of Mushroom Origin

**DOI:** 10.3390/biomedicines12071483

**Published:** 2024-07-04

**Authors:** Beata Drzewiecka, Joanna Wessely-Szponder, Michał Świeca, Paula Espinal, Ester Fusté, Eric Fernández-De La Cruz

**Affiliations:** 1 Sub-Department of Pathophysiology, Department of Preclinical Veterinary Sciences, Faculty of Veterinary Medicine, University of Life Sciences, 20-033 Lublin, Poland; beata.drzewiecka@up.lublin.pl; 2 Department of Biochemistry and Food Chemistry, University of Life Sciences, Skromna Str. 8, 20-704 Lublin, Poland; michal.swieca@up.lublin.pl; 3 Department of Pathology and Experimental Therapeutics, Faculty of Medicine and Health Sciences, University of Barcelona, 08907 Barcelona, Spain; pespinal@ub.edu (P.E.); esterfustedominguez@ub.edu (E.F.); eric.fernandez@ub.edu (E.F.-D.L.C.); 4 Department Public Health, Mental Health and Perinatal Nursing, School of Nursing, University of Barcelona, 08907 Barcelona, Spain

**Keywords:** antimicrobial peptides, bioactive peptides, fungal immunomodulatory compounds, immuno-modulation, medical mushrooms, therapeutic peptides

## Abstract

For centuries, humans have used mushrooms as both food and pro-health supplements. Mushrooms, especially those related to the functions of the human immune system, are rich in dietary fiber, minerals, essential amino acids, and various bioactive compounds and have significant health-promoting properties. Immunoregulatory compounds in mushrooms include lectins, terpenes, terpenoids, polysaccharides, and fungal immunomodulatory proteins (FIPs). The distribution of these compounds varies from one species of mushroom to another, and their immunomodulatory activities depend on the core structures and chemical modifications in the composition of the fractions. In this review, we describe active compounds from medical mushrooms. We summarize potential mechanisms for their in vitro and in vivo activities and detail approaches used in developing and applying bioactive compounds from mushrooms. Finally, we discuss applications of fungal peptides and highlight areas that require improvement before the widespread use of those compounds as therapeutic agents and explore the status of clinical studies on the immunomodulatory activities of mushrooms and their products, as well as the prospect of clinical application of AMPs as ‘drug-like’ compounds with great potential for treatment of non-healing chronic wounds and multiresistant infections.

## 1. Introduction

Nowadays, many antimicrobial agents are in common use; however, their massive and prolonged application in medicine, veterinary medicine, and agriculture has led to the emergence and spread of multidrug-resistant strains. Therefore, attention has now turned to natural compounds as innovative antimicrobials and novel immunomodulatory candidates. In recent years, there has been growing interest in immunomodulatory compounds that can support our immune system in fighting various diseases. So far, plants and microorganisms have been the main sources of these valuable compounds. However, the scientific community is increasingly focusing on exploring alternative sources. Currently, fungi are gaining particular attention as potential reservoirs of immunomodulatory compounds. There is promising evidence suggesting that certain species of fungi may contain active substances that support the immune system. Among numerous immunomodulatory products, including those of mushroom origin, antimicrobial peptides (AMPs) are attracting attention as potential candidates for biomedical and pharmaceutical applications [1]. Mushrooms have played an important role in traditional medicine and cuisine in many cultures around the world for centuries. However, in recent years, interest in their potential immunomodulating properties has grown significantly among researchers and the public. Mushroom extracts, such as reishi, shiitake, and maitake, are gaining increasing attention for their potential health benefits and ability to combat microorganisms [2]. Recently, numerous bioactive compounds have been extracted from various mushroom types. Immunomodulators aligning with the burgeoning field of immunotherapy are of particular interest. Mushroom immunomodulators, categorized by their chemical nature into lectins, terpenoids, proteins, and polysaccharides, occur naturally in greenhouse-cultivated mushrooms. To enhance industrial production efficiency, submerged cultivation is employed, elevating bioactive compound yield, reducing production time, and cutting downstream processing costs [3,4].

Another group of immunomodulatory agents are AMPs, which are low-molecular-weight peptides that have a vital function in the innate immune response of the host. These peptides are effective against a wide spectrum of microorganisms, including bacteria, fungi, parasites, and viruses. The AMP database, known as the Data Repository of Antimicrobial Peptides (DRAMP), has documented a total of 3791 AMPs from six different kingdoms. These include 431 AMPs from bacteria, 824 from plants, 7 from protozoa, 4 from archaea, 6 from fungi, and 2519 from animals [5]. These peptides mostly act by compromising the integrity of microbial cell membranes or interfering with their crucial functions, and different cells and tissues produce them in response to infection or injury. AMPs have attracted significant attention in recent years due to their potential as alternatives to conventional antibiotics, which are facing increasing challenges from antibiotic resistance. Furthermore, research on AMPs has also revealed their potential therapeutic applications in wound healing, anti-cancer treatment, and immunomodulation [6].

In this review, we discussed the immunomodulatory properties of different active compounds from mushrooms. We described their structure, potential mechanisms for their activities and potential for medical application, highlighting limitations and the need for detailed research before introduction into clinical trials.

## 2. The Host Immune Response to Stimulators

The immune system comprises a complex arrangement of cells, tissues, and organs that collaborate to protect the body from external threats. The network is interconnected through lymphatic vessels, facilitating the transport of fluid and immune cells between organs. This system comprises defensive barriers that continuously interact with lymphatic fluid abundant in white blood cells. Once pathogens breach the physical barriers, such as the skin and mucosal membranes in the mouth, nose, gastrointestinal system, and urogenital tract, the body’s adaptive immunity mechanism is triggered. The immune mechanism comprises granulocytes and monocytes, which also serve as antigen-presenting cells (APCs) for helper T lymphocytes. These cells produce and release lipid mediators, such as prostaglandins and cytokines, which function as messengers in controlling immune responses and promoting adaptive immunity. For instance, natural killer (NK) cells have the ability to identify and eliminate contaminated and aberrant cells, including cancer cells, through the initiation of apoptosis or the secretion of cytokines such as interferon-gamma (IFN-γ). In addition, they stimulate the activation of macrophages and eliminate engulfed microorganisms through phagocytosis. It is widely recognized that the human immune system can be influenced by several factors, such as dietary components, nutritional supplements, or naturally occurring bioactive substances [7].

## 3. Immunomodulatory Compounds Obtained from Mushrooms

One notable source of natural immunomodulators is medicinal mushrooms (MMs). MMs are a subset of all mushrooms and are often characterized as macroscopic fungi utilized for therapeutic purposes in the form of extracts or entire powdered mushrooms [8]. This approach could promote a balanced, healthy diet. Additionally, it could aid in the prevention, diagnosis, and treatment of human diseases [9]. Biomass or specific extracts from all stages of MM development, including fruiting bodies, mycelium, sclerotium and spores are used as dietary supplements or health foods [4].

In medicinal mushrooms, the primary categories of chemicals that possess immunomodulatory effects include terpenes and terpenoids, polysaccharides (specifically β-d-glucans, as well as polysaccharopeptides and polysaccharide proteins), lectins, and fungal immunomodulatory proteins (FIPs) [10].

Understanding the different categories of immunomodulatory compounds found in medicinal mushrooms can provide valuable insights into their potential applications in promoting tissue regeneration and maintaining immunological balance [10,11].

Various immunoregulatory substances have been extracted from therapeutic mushrooms, such as mushroom fruiting bodies and fermented mycelia. In vitro studies have shown that various mushrooms have chemo-preventive and anti-inflammatory potential. For instance, two distinct polysaccharides derived from mushroom species have demonstrated noteworthy immunoenhancing properties. Initially, a substance called glucuronoxylomannan TAP-3 was derived from *Naematelia aurantialba* (also known as *Tremella aurantialba*). This substance demonstrated significant immune-boosting properties, stimulating the release of nitric oxide (NO), interleukin-1β (IL-1β), and tumor necrosis factor-alpha (TNF-α) from macrophages [12]. Another study demonstrated that when present at a concentration of 40 μg/mL, *Craterellus cornucopioides* (L.) Pers. Polysaccharide (CCP) derived from black trumpet (*Craterellus cornucopioides*) enhanced the ability of macrophages to engulf and destroy foreign particles, increased the production of signaling molecules called cytokines, and stimulated the expression of a cell membrane receptor called TLR4 and its associated protein kinase products by activating the TLR4-NFκB pathway [13]. Certain bioactive substances have the ability to directly target cancer cells and exhibit immunoregulatory effects. Li et al. found that the polysaccharide LRP-1, which was extracted from wrinkled leccinum (*Leccinum rugosiceps*), hindered the growth of HepG2 cells (human hepatoma cells) and MCF-7 cells (human breast cancer cells). Additionally, it stimulates the release of NO, interleukin-6 (IL-6), and TNF-α in vitro [14]. Moreover, a recent study demonstrated that an aqueous extract derived from shingled hedgehog (*Sarcodon imbricatus*) successfully suppressed the proliferation, movement, and invasive characteristics of breast cancer cells in vitro and also resulted in reduced tumor growth in living organisms. Additionally, this extract caused enhanced expression of PD-L1 and improved viability of NK cells [15]. In addition, Xue et al. discovered that a triterpenoid called EAe, derived from the king trumpet mushroom (*Pleurotus eryngii*), effectively suppressed the proliferation of the MCF-7 cell line with an EC50 value of 298 μg/mL. Furthermore, it demonstrated a strong dose-dependent inhibition of CD-1 tumors in mice, achieving a 65% inhibition rate without any observed toxicity to healthy tissues [16]. The ethyl acetate extracts from mushrooms showed higher inhibitory activity against COX-1 and COX-2 enzymes than 70% ethanolic extracts. Additionally, extracts from *Ganoderma applanata*, *Naematoloma sublateritium*, *Pleurotus eryngii*, and *Pleurotus salmoneostramineus* showed higher COX-2 inhibitory effects. Ergosterol peroxide and ergosterol from edible or medicinal mushrooms suppress LPS-induced inflammatory responses by inhibiting NF-κB and CCAAT/enhancer-binding protein beta transcriptional activity and phosphorylation of MAKs [17,18].

These findings suggest that mushrooms could be used as a natural alternative to conventional anti-inflammatory drugs. The ability of certain mushrooms to specifically target COX-2 enzymes makes them particularly promising in the treatment of inflammatory stress. Furthermore, the identified compounds in mushrooms offer a potential mechanism for their anti-inflammatory effects, providing a scientific basis for their traditional use in reducing inflammation and pain [2].

### 3.1. Terpenes and Terpenoids

Terpenes and terpenoids (Table 1) are organic compounds found in medicinal mushrooms that have been shown to have immunomodulatory effects. These compounds can help regulate the immune system by either enhancing or suppressing its activity, depending on the specific needs of the body.

**Figure 1:**
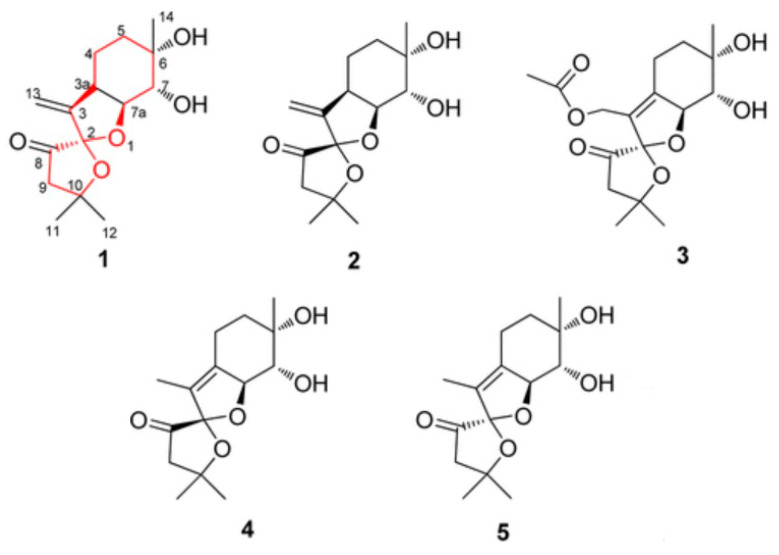
Pleurospiroketals A–E.

**Figure 2:**
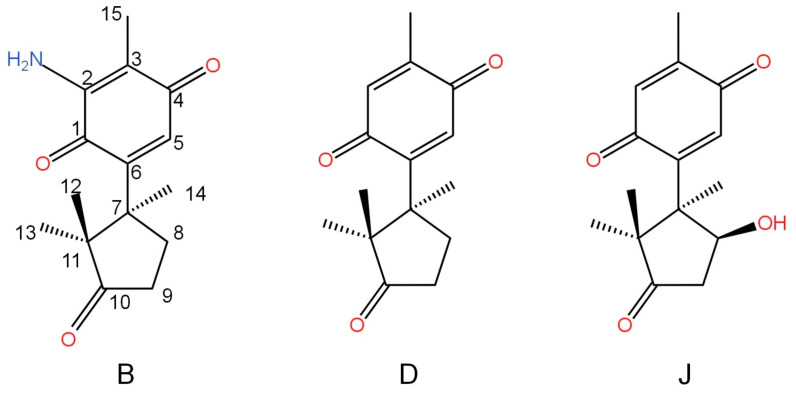
Enokipodins B, D, J.

Terpenes are a vast and varied group of hydrocarbon molecules that are produced by the biosynthesis of isopentenyl pyrophosphate units. They have a wide distribution in the natural world and are generated by various plants, including some insects, conifers, and fungi, such as mushrooms. Terpenoids are formed by the introduction of functional groups, often those containing oxygen, to terpenes. The immunoregulatory actions of both terpenes and terpenoids derived from several medicinal mushrooms have demonstrated significant medical importance. For instance, *Ganoderma* sp. is renowned for its abundant triterpenoid content, which has demonstrated potent immunomodulatory and anti-infective properties. Previously, terpenes and terpenoids were estimated to regulate immune system function by influencing the transcription of genes encoding proteins in the nuclear factor-kappa B (NF-κB) pathway and mitogen-activated protein kinase (MAPK) pathways [28,29,30]. According to Prastiyanto et al. [31], thin-layer chromatography analysis of *Pleurotus* species indicated the presence of terpenoids. *P. ostreatus* exhibits promising properties as an anti-cancer and anti-bacterial agent, specifically targeting Raji cells and methicillin-resistant *Staphylococcus aureus* (*MRSA*) strains. Previous research has demonstrated that terpenes exhibit strong antioxidant, anti-tumor, anti-inflammatory, antiviral, anti-cytotoxicity, insecticidal, and nematicidal properties [32,33,34,35]. Five unique 5,5-spiroketal sesquiterpenes, called pleurospiroketals A–E (1–5) (Figure 1), were extracted from *Pleurotus cornucopiae* edible mushroom culture and exhibited inhibition of HeLA cell proliferation [19]. Enokipodins B, D and J (Figure 2) extracted from *Flammulina velutipes* showed antioxidant activity in a DPPH scavenging assay and moderate cytotoxicity against human tumor cell lines (MCF-7, HepG2, A549, and SGC7901) [36].

### 3.2. Polysaccharides

Polysaccharides (Table 2), such as β-d-glucans, polysaccharopeptides, and polysaccharide proteins, are complex carbohydrates found in MMs that have been shown to have immunomodulatory properties. Polysaccharides are complex carbohydrates composed of monosaccharide units that are linked together by glycosidic bonds. Medical mushrooms contain various types of polysaccharides, specifically those belonging to the β-glucan, proteoglycan, and heteroglycan families. These polysaccharides can stimulate various components of the immune system and enhance its overall function [11,37].

Exopolysaccharides (EPS) are complex polymers composed of sugar molecules. They are produced by a variety of microorganisms, particularly mushrooms, and are known for their diverse biological and pharmacological properties [48]. Under typical circumstances, cells release exopolysaccharides into the surrounding medium. The functions of EPS in mushrooms include adhering to the substrate, immobilizing exocellular enzymes, preventing hyphal dehydration, and storing additional nutrients [48,49].

Homoglycans are polysaccharides that consist only of monosaccharide residues of a single kind, as per their description. Heteroglycans are polysaccharides composed of many types of monosaccharide monomers [50,51]. Glucan is a type of polysaccharide, which is a complex carbohydrate made up of several sugar units. It can have many types of links between these units, such as (1→3), (1→6)-β-glucan, and (1→3)-α-glucans. These diverse types of glucans are found in mushrooms and have the ability to modulate the immune system, making them biological response modifiers (BRMs). BRMs are classified into two categories based on their effects on cytokine and immunomodulatory responses [52,53,54].

Out of all the numerous kinds of polysaccharides found in mushrooms, β-D-glucans are thought to be the most significant ones that modulate immunity. β-D-glucan is a repeating structure made up of β-glycosidic links connecting d-glucose units. Numerous β-D-glucans, both linear and branched, have been documented thus far, exhibiting a vast array of bioactivities [37]. Beta-glucan exhibits immune-modulatory characteristics, including the ability to resist infections and exert anti-neoplastic effects on both malignant and benign tumors. The bioactive polysaccharide synthesized by *P. ostreatus* can frequently be obtained from the mycelia of the species without the necessity of waiting for the fruit body to fully mature. As a result, mycelia cultivation has gained significant interest as an effective approach for industrially producing valuable bioactive compounds and diverse agroindustrial by-products. This culture method has also been attempted as a less expensive alternative growth medium for cultivating this type of mushroom [50,55].

The primary application of β-glucans derived from *P. ostreatus* is as an adjuvant for anti-tumor therapy. The majority of cancer patients have been progressively incorporating these fungi into their therapy regimen as dietary supplements. β-glucans with higher molecular weight, such as pleuran, seem to directly stimulate leucocytes. The immune system carries out phagocytic, cytotoxic, and anti-microbial functions by producing reactive intermediates, cytokines, pro-inflammatory mediators, and chemokines. These molecules are triggered when cell surface receptors recognize pleuran molecules. β-glucans have a crucial function in stimulating T-helper lymphocyte 1 (Th1) and T-helper lymphocyte 2 (Th2), which are important types of helper lymphocytes. Th1 cells govern the intracellular immune response, while Th2 lymphocytes are responsible for the immunological defense against external infections. Water-soluble polysaccharides derived from *Pleurotus citrinopileatus* were administered to mice, leading to a notable augmentation in the population of T helper cells. Lymphocytes are known to secrete a wide range of cytokines. Th1 cells generated interferon gamma (IFN-γ) and interleukin 2 (IL-2), whereas Th2 lymphocytes secreted interleukin 4, 5, and 6 [45,54].

Pleurans are β-(1,3/1,6)-D-glucans present in several fungal species. Pleuran is a polysaccharide composed mainly of β-glucose molecules. It has a chemical formula of (C_6_H_12_O_6_)n and a molecular weight of 762 kDa. The carbohydrate content consists of D-galactose and D-glucose, which have been determined using gas chromatography. The ratio of D-galactose to D-glucose is 2:1. The primary structures of pleuran consist of a triple helix coil, which is connected to single or double filaments of glucopyranoses [45,56,57]. According to this research, it was also mentioned that a triple helix structure will emerge when the C2-position is joined with 3-H. The side chains play a role in stabilizing the conformation. A triple helix structure can form in beta-glucans that have a molecular weight exceeding 90 kDa. Swathi et al. [58] conducted a prior experiment on the characteristics of pleuran isolated from *Pleurotus ostreatus*. The findings indicate that pleuran exerts have a beneficial impact on antioxidant activity and concurrently diminish precancerous lesions in the colon of rats. Pleuran derived from *P. ostreatus* and lentinan extracted from *L. edodes* are among the polysaccharides that are extensively employed in various industries [56].

### 3.3. Proteins and Peptides from Mushrooms—Mushroom Bioactive Peptides (MBP)

Mushroom proteins and protein-conjugate complexes are recognized for their immunomodulatory properties. Protein-based immunomodulatory substances found in MMs can be classified into two primary categories: lectins and FIPs. FIPs are distinguished from lectins by their lack of conjugates, whereas each lectin consists of unique carbohydrates that are linked to a polypeptide [11].

#### 3.3.1. Lectins

Lectins, which are carbohydrate-binding proteins, also play a role in immunomodulation by binding to specific receptors on immune cells and influencing their activity. Lectins can be found in animals, plants, and microbes. They have the ability to link cells together by using specialized sugar-binding sites for polysaccharides and glycoconjugates. Medicinal mushroom species containing lectins (Table 3) are highly diverse and include *Floccularia luteovirens* (also known as *Armillaria luteovirens*), *Grifola frondosa*, *Ganoderma capense*, *Pseudosperma umbrinellum* (also known as *Inocybe umbrinella*), *Pleurotus citrinopileatus*, *Pholiota adipose*, *Russula delica*, *S. commune*, *Leucocalocybe mongolica* (also known as *Tricholoma mongolicum*), *Xerocomus spadiceus* and *Volvariella volvacea*. Furthermore, lectins can also be extracted from some commonly eaten mushrooms, like *Agaricus bisporus*, *Hericium erinaceum*, *Pleurotus ostreatus*, *Flammulina velutipes*, *Ganoderma lucidum*, and *Volvariella volvacea* [59].

Lectins can be found in various parts of mushrooms, including the caps, spores, stipes, and mycelia. The composition of lectin may vary depending on the carpophore’s age and the specific time and place of its collection. Mushroom lectins have significant involvement in dormancy, development, morphogenesis, and the resulting morphological alterations caused by parasitic infections. Additionally, they contribute to molecular identification during the initial phases of mycorrhization. Mushroom lectins have been found to have a variety of beneficial effects that can be utilized, such as modulating the immune system, inhibiting cell growth, and promoting cell division as an anti-tumor activity. Lectins, among other effects, have demonstrated the ability to induce the formation of nitrite, increase expression of TNF-α and interleukins, activate lymphocytes, and enhance the production of macrophage-activating factors. Furthermore, many mushroom lectins have demonstrated significant antiviral, mitogenic, antibacterial, and antioxidant properties [59,68,69,70].

#### 3.3.2. Fungal Immunomodulatory Proteins (FIPs)

FIPs are biomolecules found in MMs that can modulate immune responses (Table 4). These proteins can interact with immune cells and regulate their function. These peptides have been evaluated for their potential anti-neoplastic or anti-allergy activity and stimulation of immune cells to produce cytokines. FIPs can be used in pharmaceuticals or vaccines to enhance immune responses and suppress tumors and autoimmune diseases [71].

One of the most well-known FIPs is Ling-Zhi-8, derived from *Ganoderma lucidum*, with immunosuppressive functions, such as suppression of cell growth and proliferation, the initiation of apoptosis and autophagy, and the attenuation of tumor cell invasion and migration. Thus, apart from their immunomodulatory effects, they have demonstrated anticancer properties. Currently, the majority of these studies are performed utilizing tissue cultures. Additional experimentation on animal models and clinical trials are necessary to validate their safety and effectiveness in humans. If verified, these FIPs could be generated and commercialized more effectively through genetic engineering for therapeutic applications [3].

Li et al. [77] investigated the effects of a manufactured protein, recombinant FIP-gsi. This protein triggered the creation of instructions (gene expression) for several immune system messengers (cytokines) in mouse spleen cells. These messengers included IL-2, IL-3, IL-4, IFN-γ, TNF-α, and the IL-2 receptor. Interestingly, the protein mainly influenced two messengers from a specific type of immune cell (Th1 cells) and one messenger from another type (Th2 cells). This finding aligns with previous research on a similar protein (FIP-vvo), which also boosted the production of instructions for similar immune cytokines. Overall, the results suggest that recombinant FIP-gsi primarily targets Th1 cells and has a weaker effect on Th2 cells.

In another study, Li et al. [74] discovered a new protein, Basidiomycota D. squalens FIP-dsq2, by sequence similarity search. In comparison to the published FIPs, FIP-dsq2 showed significant sequence and structural conservativeness. On the other hand, FIP-dsq2 diverged significantly from the other FIPs in terms of phylogeny. Furthermore, FIP-dsq2 was prepared on a wide scale using recombinant expression with a GST tag in E. coli. Additionally, rFIP-dsq2 demonstrated pronounced anti-cancer properties on A549 cells, including suppression of proliferation, induction of apoptosis, and inhibition of migration. Based on these findings, it appears that rFIP-dsq2 has potential as an antitumor agent.

#### 3.3.3. Fungal AMPs

Fungal AMPs can be categorized into two groups: peptaibols, which are found in *Trichoderma* spp. and defensins, which are found in *Pseudoplectania*, *Coprinopsis*, and *Microsporum* spp. [78]. Several fungal AMPs exhibit inhibitory effects on pathogenic fungi, such as *Aspergillus* and *Candida* spp. in humans, as well as yeast and filamentous fungi (e.g., *Aspergillus flavus*), which impact food and agriculture [79]. Fungal AMPs are short peptides, consisting of 5 to 21 amino acids, and often contain a significant amount of non-proteinogenic amino acids, such as α-aminoisobutyric acid. These peptides typically include an acylated N-terminal residue and an amino alcohol, like phenylalaninol or leucenol, linked to the C-terminal [80].

An antifungal peptide called pleurostrin has been obtained from the oyster mushroom *Pleurotus ostreatus*. In terms of N-terminal sequence similarity, ganodermin is similar to two antifungal proteins found in mushrooms: Lyophyllum antifungal protein and eryngin, and, to a lesser extent, angiosperm thaumatin-like proteins and thaumatin. There are no similarities to other antifungal proteins found in mushrooms, though. In the phytopathogenic fungi *B. cinerea*, *F. oxysporum*, and *P. piricola*, ganodermin suppresses the growth of the mycelium. *P. piricola* and *M. arachidicola* are inhibited by the antifungal protein found in lyophyllum [81].

### 3.4. Different Activities of MBP

**Figure 3:**
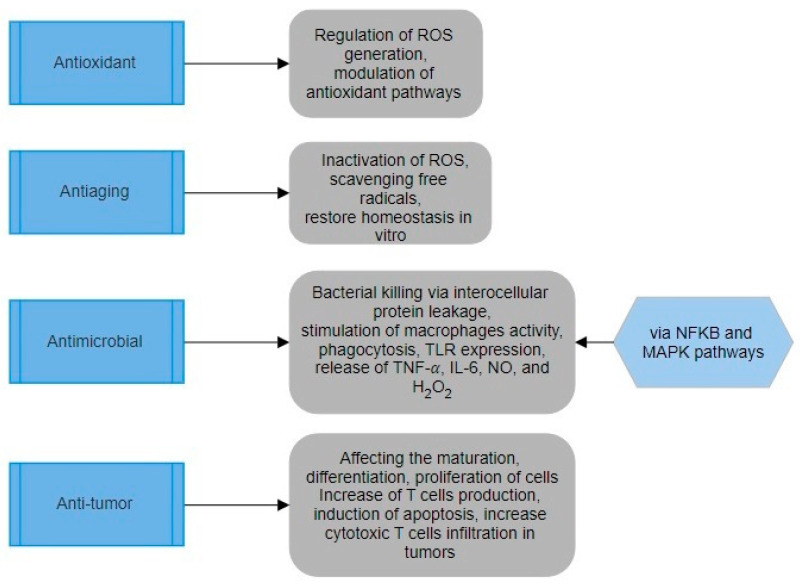
Mechanisms of action of the MBP.

Here, we discuss some different modes of action peptides from mushrooms as a promising source of pro-health food and other compounds. Figure 3 shows the main mechanisms of action of the MBP.

#### 3.4.1. Antioxidant Properties

Edible mushrooms contain many peptides that have antioxidant properties [82]. MBPs may exhibit varying targeting functionalities due to distinct basic components and production methods. MBPs primarily function in antioxidant processes by regulating reactive oxygen species (ROS) generation and antioxidant activity. MBPs can scavenge free radicals by supplying protons, electrons, and chelating metal ions to control the generation of ROS [83]. Peptides extracted from *Agaricus bisporus* (ABP) and *Pleurotus eryngii* mycelium (PEMP) contained a high concentration of negatively charged amino acids. These amino acids have the ability to counteract free radicals and control the generation of ROS [84]. *Ganoderma lucidum* peptide (GLP) demonstrated antioxidant properties in soybean oil by inhibiting soybean lipoxygenase activity in a dose-dependent manner, exhibiting an IC50 value of 27.1 µg/mL. GLP exhibited superior antioxidant activity over butylated hydroxytoluene by neutralizing hydroxy radicals and quenching superoxide radical anions in biological systems [85].

Organisms can control the generation of ROS over time via internal enzymes and non-enzymatic defense mechanisms [86]. Administering GLP orally showed strong hepatoprotective effects in rats with liver damage due to its antioxidant activity [87]. GLP elevated glutathione (GSH) and superoxide dismutase (SOD) levels while reducing malondialdehyde (MDA) levels in the liver. It also reduces the levels of alanine transaminase (ALT) and aspartate transaminase (AST) in the blood to combat liver fibrosis and alcoholic liver damage. Studies demonstrated that GLP can efficiently reduce the risks associated with peroxide generated in mitochondria by controlling the function of antioxidant enzymes, displaying outstanding antioxidant properties [88].

MBPs exhibit antioxidant action by regulating antioxidant pathways such as the Kelch-like ECH-associated protein. 1-Nrf2 The text mentions various signaling pathways such as 2-antioxidant responsive elements (Keap1-Nrf2-ARE), mitogen-activated protein kinase (MAPK), nuclear factor-κ light chain enhancer of activated B cells (NF-κB), and phosphoinositide 3-kinase/protein kinase B (PI3K/AKT) [89,90,91]. MBPs can control the levels of antioxidant proteins by decreasing the activity of the Keapl gene and increasing the expression of the Nrf2 gene [83]. The GLP stimulated Nrf2 and triggered the Nrf2-ARE signaling cascade, demonstrating antioxidant properties in cells exposed to hydrogen peroxide (H_2_O_2_) [92]. Upon activation of the PI3K/AKT and MAPK pathways, Nrf2 separates from Keap1, translocates to the nucleus, and binds to the antioxidant component ARE, which controls the production of antioxidant enzymes including HO-1, catalase (CAT), and others [93].

Aging affects cells, organs, and the entire organism, resulting in a decline in the body’s capacity to remove oxidative stress and a decrease in biological function. Excessive free radicals lead to elevated MDA levels, decreased total antioxidant capacity (T-AOC), and disruption of cellular structure, ultimately resulting in cellular senescence and death [94]. CMP and GLP exhibited dose-dependent scavenging effects on oxygen and hydroxyl free radicals [95]. SOD is an essential antioxidant enzyme found in mitochondria that is linked to longevity [96]. Macrophage binding proteins could greatly improve the activity of antioxidant enzymes in mitochondria linked with senescence. CMP and GLP demonstrated superior hydroxyl radical scavenging capabilities compared to the specialized hydroxyl radical scavenger mannitol [97]. *Agaricus blazei* peptide (ABp) decreased MDA and ROS levels, and enhanced CAT and T-AOC functions in a D-galactose-induced aging model of NIH/3T3 cells [98].

MBPs primarily target the metabolic mitochondrial pathway, deactivate ROS, eliminate free radicals, reduce the oxidation of biomarkers in organisms, and restore homeostatic mechanisms in living creatures [99]. Nrf2 activity was closely associated with age-related degenerative disorders and played a role in both preventing and alleviating these conditions. ABp feeding in the D-galactose aging model in mice was discovered to decrease Keap1 protein expression, resulting in an increase in Nrf2 levels. Within the Keap1-Nrf2 pathway, the up-regulation of HO-1 and associated components such as ApoE, Hsph1, and Trim32 led to the efficient removal of free radicals, demonstrating strong anti-aging properties [100]. Research on aging revealed progressive alterations in epigenetic information in both proliferating and quiescent cells, including adjustments in chromatin structure, histone modifications, and DNA methylation patterns. Aging is primarily characterized by changes in DNA and histones methylation, along with other epigenetic modifications [101]. Previous research has shown that consuming peptides can directly influence the epigenetic changes associated with aging by regulating telomere length [96]. There is no current study on whether MBPs can alter telomere length in various organisms. This calls for further research on the anti-aging properties of MBPs, indicating a new research topic that requires more investigation in the future.

In conclusion, MBPs can target many sites concurrently to demonstrate their functional antioxidant and age-delaying effects. MBPs have great potential for growth as a key element in natural antioxidant and anti-aging functional foods. MBPs are now a focus of research in the field of functional foods. Additional research is necessary to investigate how MBPs can substitute for synthetic antioxidants [88].

#### 3.4.2. Antimicrobial Activity

Antibiotics are effective in treating many serious diseases like tuberculosis, pneumonia, leprosy, and gonorrhea, among others. However, antibiotic resistance has developed due to the excessive and inappropriate use of antibiotics [102,103]. Natural bioactive peptides are known for their great effectiveness, stability, and minimal toxicity, positioning them as significant substitutes for antibiotics and other medications [104]. Mushrooms have been extensively researched for their antibacterial properties. Several antimicrobial peptides have been extracted and refined from edible mushrooms such as *Polyporus alveolaris*, *Pleurotus eryngii*, *Lentinus edodes*, and *Agrocybe cylindracea* [105,106]. The potential antibacterial actions of MBPs may include altering tissue-specific expression patterns or causing intracellular protein leakage, resulting in bacterial mortality.

MBPs’ hydrophobic amino acids may control the NF-κB pathway and the MAPK pathway. PEMP exhibited potent antibacterial effects through enhancing macrophage proliferation, boosting phagocytosis activities, increasing TLRs expression, and releasing tumor necrosis factor-α (TNF-α), IL-6, NO, and H_2_O_2_ [84,106]. MBPs contain a variety of topologies, including α-helices, β-folds, random coils, and disulfide linkages. Antimicrobial peptides contain hydrophobic amino acids, β-folds, α-helixes, random coils, and disulfide bonds.

MBPs have an additional antibacterial function by disrupting bacterial cell membranes, leading to the release of intracellular proteins and ultimately achieving antibacterial effects. Antimicrobial peptides derived from the mycelia of scarlet caterpillarclub (*Cordyceps militaris*) can induce intracellular protein leakage in *Escherichia coli* (ATCC 25922), leading to the preservation of intestinal mucosa integrity and a reduction in *E. coli* infections in mice. Antibacterial peptides extracted from the mycelia (GLM) and fruiting bodies (GLF) of *G. lucidum* showed a dose-dependent rise in protein leakages from *E. coli* and *Staphylococcus aureus* at concentrations ranging from 50 to 125 μg/mL. GLF and GLM clearly trigger cell death and have potent antibacterial effects against *E. coli* and *S. aureus* [107,108]. According to the information provided, MBPs exhibit strong antibacterial properties against drug-resistant microorganisms. MBPs may serve as significant natural substitutes for antibiotics.

#### 3.4.3. Anti-Inflammatory Activity

Macrophage phagocytosis and NK cell cytotoxicity may be boosted by MBPs, leading to increased proliferation and development of immune cells and lymphocytes while suppressing pro-inflammatory reactions, thereby enhancing the host’s immunity against infections [109]. Qiuhui et al. [110] synthesized a bioactive peptide, KSPLY, from Lion main (*Hericium erinaceus*). KSPLY enhanced the secretion of TNF-α, NO, IL-6, and IL-1β by macrophages, leading to the inhibition of lipopolysaccharide (LPS)-induced inflammatory reactions at a dose of 100 μmol/L. Mice administered *Pleurotus eryngii* peptide (PEP) showed a significant decrease in splenic lymphocyte proliferation, while the serum hemolysin level in CTX-induced mice dramatically increased. This confirmed that PEP might greatly enhance the humoral immune activity of immunosuppressed mice [111].

**Figure 4:**
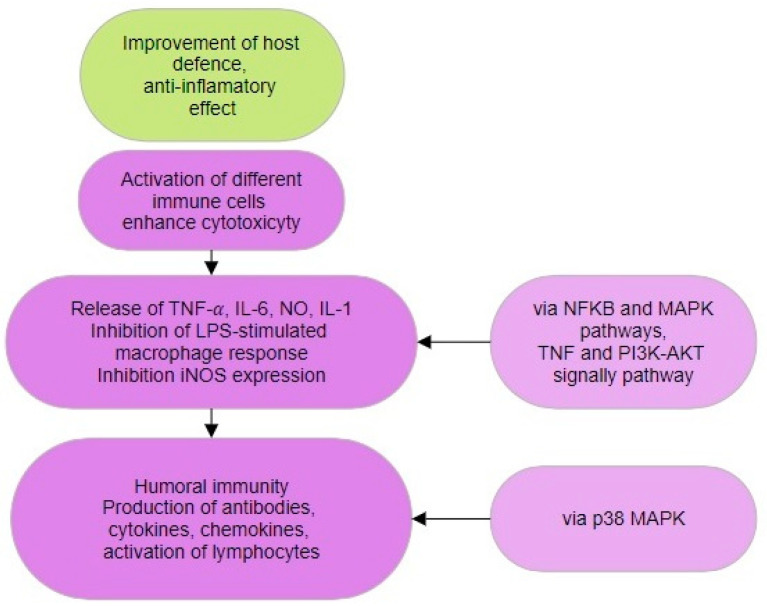
Anti-inflammatory mechanisms of MBPs activity.

MBPs can boost the antioxidant defense system and barrier function by stimulating the generation of antibodies, cytokines, and chemokines to decrease the inflammatory reaction (Figure 4). Following local nasal immunotherapy (LNIT) using winter mushroom (*Flammulina velutipes*) peptides (FIP-fve), there was a notable decrease in the production of proinflammatory cytokines and chemokines [112]. FIP-fve has been demonstrated to activate peripheral lymphocytes via stimulating the p38 mitogen-activated protein kinase (p38 MAPK) signaling pathway, resulting in anti-inflammatory effects. The peptide WFNNAGP produced from matsutake (*Tricholoma matsutake*) reduced inflammation by suppressing the production of pro-inflammatory cytokines and myeloperoxidase (MPO) activity, while also enhancing the expression of tight junction proteins such as closing ribbon-1, claudin, and occluding [113]. WFNNAGP decreased colonic inflammation in mice by suppressing NF-κB expression, therefore preventing the creation and activation of NLRP3 and caspase-1.

MBPs target several cells and sites, including NK cells, CD4+, CD25+, T lymphocytes, macrophages, monocytes, B lymphocytes, and mast cells [114,115,116]. A study showed that FIP-fve altered Treg-associated immunity by decreasing IL-4+/CD4+ T-cell levels and increasing IFN-γ+/CD4+ T-cell levels in mice. FIP-fve successfully reduced the invasion of inflammatory cells and epithelial damage [112]. Experiments showed that orally giving FIP-fve has an anti-inflammatory effect on mite-induced airway inflammation in mice. *Cordyceps militaris* bioactive polypeptides (CMP) were shown to regulate immunological function in mice by affecting transcription factors Ets1, Spp1, Rel, and Smad3 genes CMP controlled TNF and the PI3K-Akt signaling pathway, which contributed significantly to inflammation by boosting leukocyte count and hemolysin levels in mouse sera. MBPs exhibit strong anti-inflammatory properties and present novel possibilities for creating functional food supplements using natural components [117].

### 3.5. Biomedical Applications of Fungal Immunomodulatory Compounds

Capsules or tablets containing some of the nutrients derived from mushrooms, or mushroom nutraceuticals, have been fabricated as dietary supplements. Frequent use of these nutritional supplements has been linked to an improved immunological response in humans, which increases immunity against infections and enhances recovery from a variety of illnesses [118,119,120]. There are already hundreds of branded MM items available for purchase worldwide. Anticancer, immune-stimulating, antioxidant, hepatoprotective, neuroprotective, antihypertensive, antifungal, antibacterial, and antiviral properties are only a few of the health advantages of MM products [121]. Numerous elements have been linked to their effects, including minerals, proteins, dietary fiber, lipopolysaccharides, polysaccharides, secondary metabolites, and glycoproteins. Several complex chemical molecules have demonstrated immunomodulatory properties [8]. As an illustration, the polysaccharides found in MMs have the ability to stimulate neutrophils, macrophages, and natural killer cells in addition to innate interleukins and immune cytokines. Furthermore, by maintaining the stability of their critical metabolic processes, MM secondary metabolites, including phenols, terpenes, and sterols, might improve host survival [9].

In addition to phenols, terpenes, and sterols, other metabolites found in MMs have also been shown to potentially enhance host survival. These functional components can have diverse effects on immunomodulatory pathways, making the composition and purpose of MMs vary greatly. Furthermore, understanding the molecular mode of action of these functional components is crucial to comprehending their impact on the immune system [11].

## 4. Limitations

The use of preparations of mushroom origin encounters some problems and limitations. Out of over 70,000 known types of mushrooms, only around 2000 (31 genera) are classified as edible mushrooms [122]. Pollutants originating from human activities such as fuel burning, urban growth, agrochemicals, and industrial development are typically emitted into the atmosphere, soil, or water, disrupting the balance of the ecosystem [123]. Industrialization, urbanization, combustion of fuels, and use of agrochemicals are the primary causes of environmental concerns. This could result in the buildup of harmful substances such as heavy metals in soils and sediments, which may then transfer into the human food chain [124]. Heavy metals such as Cobalt (Co), Copper (Cu), Lead (Pb), Manganese (Mn), Chromium (Cr), Cadmium (Cd), Zinc (Zn), and Iron (Fe) are known to be highly dangerous pollutants [125]. Fruiting bodies of mushrooms are known to accumulate heavy metals [126]. These heavy metals are believed to cause severe toxicological harm to health, even at low concentration levels. Consuming mushrooms polluted with heavy metals can damage the kidneys and heart, resulting in the failure of several bodily systems such as the digestive, immunological, skeletal, and nervous systems [127,128].

Another problem is involved in the decomposition of the mushroom compounds. Lectins are resistant to digestion by enzymes. Significant quantities will accumulate in tissues such as the gut lining, joints, nerve junction areas, and myelin sheaths, leading to inflammation and persistent inflammatory reactions that might result in lasting damage to these tissues and locations. Legumes, nuts, and grains are known to have significant levels of bioactive lectins. However, regarding daily consumption, fungi, particularly mushrooms, are considered one of the safest foods in terms of lectin concentration [4].

## 5. Perspectives and Conclusions

The increasing prevalence of antimicrobial resistance is leading to non-healing chronic wounds, multiresistant infections, polymicrobial biofilms, and compromised wound healing. As populations age, chronic metabolic diseases and hard-to-heal wounds increase. AMPs have significant benefits over traditional antibiotics due to their antimicrobial and anti-biofilm activities, as well as their potential as broad-spectrum antibiotics. AMPs also have immunomodulatory activities and wound healing-promoting actions due to their anti-inflammatory effects and ability to drive cell proliferation and migration for tissue repair [129,130,131].

However, the instability and toxicity associated with AMPs during production and clinical use restrict their topical use. To enhance the efficacy of future AMPs, chemical modifications, target-based rationalized approaches, and synthesis are needed. Carriers and nanoformulation strategies that protect AMPs and provide controlled release are needed to expose their optimal activities as ‘drug-like’ compounds. Currently available smart delivery nanocarriers provide an effective way to stabilize and protect peptides against enzymatic degradation. However, the effectiveness of novel nanoformulation strategies is limited by production techniques, harsh solvents, the physical and chemical degradation of peptides, and cytotoxicity at higher doses. To address these challenges, it is necessary to choose the appropriate composition and manufacturing strategies for AMPs during the formulation step [129].

This article discussed the immunomodulating properties of various mushroom components and investigated how they work. It is anticipated that a combination of these pathways will be identified as responsible for the anti-inflammatory effects of mushrooms. Most mushroom studies primarily examine testing using crude extracts of either the whole mushroom fruiting bodies or mycelia. In the future, it is necessary to utilize purified bioactive chemicals extracted from mushrooms in investigations involving animals and humans. Additional research is needed to elucidate the effects and relationships between these bioactive compounds and other dietary elements.

## 6. Summary

The functional components of various MMs as well as natural blood-derived AMPs vary and may have variable effects on the same or distinct immunomodulatory pathways. We gave a quick overview of the components documented in MMs and linked them with immunomodulatory compounds from animal blood. The variety, composition, purpose, and molecular mode of action of the functional components in MMs and AMPs that have been demonstrated to be involved in immune system modulation are then briefly summarized. Finally, we conclude with a brief discussion of how different kinds of mushroom substances may expedite studies on new forms of therapeutic products.

## Figures and Tables

**Table 1:** Immunomodulatory terpenes and terpenoids from medicinal mushrooms.

Source	Immunomodulating Compounds	Model	Reference
*Pleurotus cornucopiae*	Pleurospiroketals A, B, C	HeLa cells	[19]
*Flammulina velutipes*	Enokipodins B, D, J	Hela, HepG2, and KB cell lines	[20]
*Stereum hirsutum*	Sterehirsutynes A–C	porcine pancreatic lipase (PPL)	[21]
*Cyathus africanus*	Cyathins Q–X	-	[22]
*Sarcodon scabrosus*	Sarcodonin A	C57BL/6 mice brain cells	[23]
*Hericium erinaceus*	Erinacine A	Rat model of ischemia	[24]
*Cyathus striatus*	Striatals A–D, striatoids A–F	MCF7 breast cancer cells	[24]
*Leucopaxillus gentianeus*	Cucurbitacin D, 16-deoxycucurbitacin B	NCI-H460 human tumor cell line	[25]
*Hexagonia tenuis*	Hexagonin F	SK-LU-1, HepG2, Hep3B, SW480, and MCF-7 tumor cell lines	[26]
*Humphreya endertii*	Endertiins A–B	MCF7 (human breast carcinoma) and LU (human lung carcinoma) cell lines	[27]

**Table 2:** Immunomodulatory polysaccharides from medicinal mushrooms.

Source	Immunomodulating Compounds	Model	Reference
*Agaricus blazei*	Heteroglycan, Glycoprotein, Glucomannan-protein complex, β-1,3-d-glucan, with β-1,6-d-glucan branch	KYSE150 and KYSE170 cells	[38]
*Auricularia auricula-judae*	Mannose, xylose, glucuronic acid, and glucose. The molecule contained α-Glc(1→4)-, β-Glc(1→3)-, and β-Man(1→4)-linked glycosidic bonds	Male ICR mice	[39]
*Flammulina velutipes*	*Flammulina velutipes* peptidoglycan (FVP)	High-fat diet-fed (HFD-fed) obese mice	[40]
*Grifola frondosa*	Grifola frondosa polysaccharide (GFP) with β-1,3-linked or β-1,6-linked glucan structure	Heps-bearing mice	[41]
*Ganoderma lucidum*	GLP-1 and GLP-2	Kunming mice (SPF, male)	[42]
*Lentinula edodes*	Three polysaccharide fractions (F1, F2 and F3)	Female BALB/c mice	[43]
*Morchella esculenta*	Morchella esculenta Polysaccharide (MCP), β-1,3-d-glucan	Kunming mice	[44]
*Pleurotus ostreatus*	β-Glucan	Intestinal epithelial cell line HT-29, the HEK-Blue™hTLR4 cell line	[45]
*Phellinus linteus*	Heteropolysaccharide PL-N1	HepG2 cell	[46]
*Xylaria nigripes*	β-Glucan	Macrophage cells of Balb/c mice	[47]

**Table 3:** Immunomodulatory lectins from medicinal mushrooms.

Source	Immunomodulating Compounds	Model	References
*Agaricus bitorquis*	Agaricus bisporus lectin (ABL)	Human liver cancer cell line Hep G2 and mouse lymphocytic leukemia cell line L1210	[60]
*Phellodon melaleucus*	Phellodon melaleucus lectin (PML)	B16 melanoma mouse model	[61]
*Agrocybe aegerita*	AAL	Breast cancer, cell line 4 T1	[62]
*Marasmius oreades*	MOA	SKBR3 cell line	[63]
*Boletus edulis*	BEL	HepG2 cell line	[64]
*Psathyrella asperospora*	PAL	Colon cancer HT29 cell line	[65]
*Lignosus rhinocerotis*	LRL	HeLa, MCF7 and A549 cells	[66]
*Clitocybe nebularis*	CNL	CRL8066 cell line	[65]
*Boletus speciosus*	BSH	HepG2 and L1210 cells	[67]
*Lignosus rhinocerotis*	FIP-Lrh	Lung cancer A549 cell line	[65]

**Table 4:** Immunomodulatory FIPs from medicinal mushrooms.

Source	Immunomodulating Compounds	Model	Reference
*Morchella conica*	FIP-mco	Cell lines (A549, HepG2, and THP-1)	[72]
*Chroogomphis rutilus*	FIP-cru1	Murine splenocytes	[73]
*Dichomitus squalens*	FIP-dsq	A549 cells	[74]
*Ganoderma lucidum*	FIP-glu2	THP-1 macrophages	[71]
*Lignosus rhinocerotis*	FIP-Irh	MCF7, HeLa and A549 cancer cell lines	[66]
*Ganoderma microsporum*	GMI	A549 cells	[75]
*Flammulina velutipes*	FIP-fve	human peripheral lymphocytes	[11]
*Trametes versicolor*	FIP-tve2	human peripheral blood lymphocytes	[11]
*Volvariella volvacea*	FIP-wo	-	[76]
*Lentinus tigrinus*	FIP-lti1, FIP-lti2	-	[76]

## Data Availability

Data is contained within the article.
